# Autosomal recessive variants in *TUBGCP2* alter the γ-tubulin ring complex leading to neurodevelopmental disease

**DOI:** 10.1016/j.isci.2020.101948

**Published:** 2020-12-30

**Authors:** Serdal Gungor, Yavuz Oktay, Semra Hiz, Álvaro Aranguren-Ibáñez, Ipek Kalafatcilar, Ahmet Yaramis, Ezgi Karaca, Uluc Yis, Ece Sonmezler, Burcu Ekinci, Mahmut Aslan, Elmasnur Yilmaz, Bilge Özgör, Sunitha Balaraju, Nora Szabo, Steven Laurie, Sergi Beltran, Daniel G. MacArthur, Denisa Hathazi, Ana Töpf, Andreas Roos, Hanns Lochmuller, Isabelle Vernos, Rita Horvath

**Affiliations:** 1 Inonu University, Faculty of Medicine, Turgut Ozal Research Center, Department of Paediatric Neurology, Malatya, Turkey; 2 Izmir Biomedicine and Genome Center, Dokuz Eylul University Health Campus, Izmir, Turkey; 3 Department of Medical Biology, Faculty of Medicine, Dokuz Eylul University and Izmir International Biomedicine and Genome Institute, Dokuz Eylul University, Izmir, Turkey; 4 Dokuz Eylul University, Faculty of Medicine, Department of Pediatric Neurology Izmir, Turkey; 5 Centre for Genomic Regulation (CRG), the Barcelona Institute of Science and Technology, Dr. Aiguader 88, Barcelona 08003, Spain; 6 Pediatric Neurology Clinic, Private Office, Diyarbakir, Turkey; 7 John Walton Muscular Dystrophy Research Centre, Institute of Translational and Clinical Research, Newcastle University, Newcastle upon Tyne, UK; 8 Department of Clinical Neurosciences, John Van Geest Cambridge Centre for Brain Repair, University of Cambridge School of Clinical Medicine, Robinson Way, Cambridge CB2 0PY, UK; 9 Budai Children Hospital, Észak-Közép-budai Centrum, Új Szent János Kórház és Szakrendelő, Budapest, Hungary; 10 CNAG-CRG, Centre for Genomic Regulation, Barcelona Institute of Science and Technology, Barcelona, Spain; 11 Analytic and Translational Genetics Unit, Massachusetts General Hospital, Boston, MA, USA; 12 Program in Medical and Population Genetics, Broad Institute of MIT and Harvard, Cambridge, MA, USA; 13 Leibniz Institut für Analytische Wissenschaften, ISAS, Dortmund, Germany & Pediatric Neurology, University Hospital, University of Duisburg-Essen, Faculty of Medicine, Essen, Germany; 14 Children's Hospital of Eastern Ontario Research Institute; Division of Neurology, Department of Medicine, the Ottawa Hospital; and Brain and Mind Research Institute, University of Ottawa, Ottawa, Canada; 15 Universitat Pompeu Fabra (UPF), Barcelona, Spain; 16 Institució Catalana de Recerca i Estudis Avançats (ICREA), Spain

**Keywords:** Biological Sciences, Neuroscience, Molecular Neuroscience, Clinical Neuroscience, Systems Biology, Protemics

## Abstract

Microtubules help building the cytoskeleton of neurons and other cells. Several components of the gamma-tubulin (γ-tubulin) complex have been previously reported in human neurodevelopmental diseases. We describe two siblings from a consanguineous Turkish family with dysmorphic features, developmental delay, brain malformation, and epilepsy carrying a homozygous mutation (p.Glu311Lys) in *TUBGCP2* encoding the γ-tubulin complex 2 (GCP2) protein. This variant is predicted to disrupt the electrostatic interaction of GCP2 with GCP3. In primary fibroblasts carrying the variant, we observed a faint delocalization of γ-tubulin during the cell cycle but normal GCP2 protein levels. Through mass spectrometry, we observed dysregulation of multiple proteins involved in the assembly and organization of the cytoskeleton and the extracellular matrix, controlling cellular adhesion and of proteins crucial for neuronal homeostasis including axon guidance. In summary, our functional and proteomic studies link TUBGCP2 and the γ-tubulin complex to the development of the central nervous system in humans.

## Highlights

• *TUBGCP2* variants cause neurodevelopmental delay, brain malformation, and epilepsy
• The variant alters GCP2/GCP3 interaction and localization of GCP2 in cell cycle
• We link GCP2 to the cytoskeleton, extracellular matrix, cell adhesion, and axon guidance
• Functional proteomics is useful in establishing molecular pathways in rare diseases

## Graphical Abstract

**Figure:**
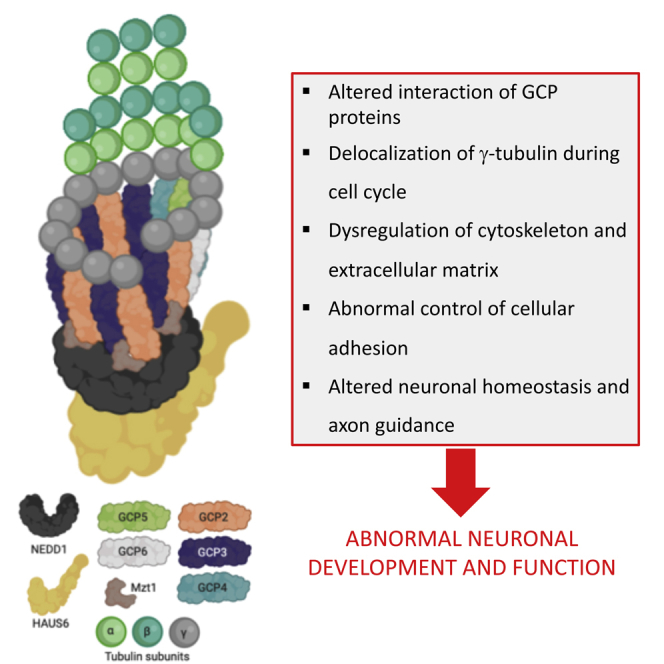

## Introduction

Microtubules (MTs) are dynamic, cytoskeletal polymers crucial for cortical development and neuronal migration. Mutations in several genes encoding alpha-tubulin (*TUBA1A*), beta-tubulin (*TUBB2A*, *TUBB2B*, *TUBB3*, *TUBB4A*, *TUBB*), and gamma-tubulin (γ-tubulin) (*TUBG1*) isoforms have been associated with a wide range of brain malformations including lissencephaly, polymicrogyria, microlissencephaly, and simplified gyration (Romaniello et al., 2018). Mutations in different tubulin genes cause various phenotypes (Table 1). Alpha-tubulin and γ-tubulin gene mutations predominantly result in lissencephaly spectrum diseases (Romaniello et al., 2018). Beta-tubulin gene mutations may show normal cortical pattern; however, *TUBB4A* is predominantly associated with hypomyelination and cerebellar and brainstem atrophy (Blumkin et al., 2014). *TUBB2B* and *TUBB3* mutations seem to be more related to polymicroglial patterns. Microcephaly and ocular malformations are commonly seen in beta-tubulin (TUBB) defects (Francis and Belvindrah, 2018; Romaniello et al., 2018).

**Table 1:** Functional effect and clinical phentotype of pathogenic mutations in tubulin complex protein genes

Function	Gene	Variants (nucleotide/protein/zygosity)		Effects		Severity	Clinical features	Common MRI findings (# of positive cases/ # of total cases)
ɤ-TuSC and ɤ-TuRC genes (except ɤ-tubulins)	TUBGCP2	c.1015G > A, p.Glu311Lys, Hom		Changes in TUBGCP2, HAUS6, NEDD1 protein localizations in mitosis/no change in GCP2 level		Severe or moderate	DD ID Facial dysmorphism Hypotonia	Pachygyria (7/7) Thin CC (6/7) Cerebellar atrophy(5/7) WM volume loss (3/7) Brainstem atrophy (2/7) Subcrotical band (2/7) WM hyperintensity with subependimal cysts (4/7)
		c.997C > T, p.Arg333Cys, Hom^a^		Alteration in the part of the conserved Grip1 domain		Severe (2q23.1 dup) or moderate		
		c.1843G > C, p.Ala615Pro, Hom^a^		Changes in the Grip2 domain		Severe		
		c.889C > T, p.Arg297Cys c.2025-2A > G, Cmp Het^a^		Changes in the extended conserved Grip1 domain		Mild		
				Alternative splice acceptor site; excision of exon 15 or inclusion of intron 13 and premature stop codon				
	TUBGCP4	c.1746G > T, p.Leu582 =,Cmp Het^b^		Alternative splice acceptor site; exon 16 skipping	Truncated GCP4 protein and reduced amounts of GCP4 and other proteins; GCP2, GCP5, GCP6, ɤ-tubulin in interphase and in mitosis, reduced levels of ɤ-TuRC		Congenital microcephaly Chorioretinopathy (MCCRP) ID Facial dysmorphism	Thin CC (1/5) Normal (4/5) No cortical malformation
		*c.1746G > T*	+ c.579dupT, p.Gly194Trpfs∗8^b^	Frameshift mutation		Moderate (thin CC and ID)		
		*c.1746G > T*	+ c.298delT, p.Tyr100Ilefs∗27^b^	Frameshift mutation		Mild		
		*c.1746G > T*	+ c.1732-?_∗544+?del^b^	Exon 16-18 del, ~544 nucleotide del of 3′ UTR		Mild		
		*c.1746G > T*	+ c.1380G > A, p.Trp460∗^c^	Nonsense mutation		Mild		
	TUBGCP5	c.2180T > G p.Phe727Cys with 15q11.2 BP1-BP2 microdeletion^d^		Missense variant		Mild	Primary microcephaly DD	No cortical malformation Normal
	TUBGCP6	c.2066-6A > G, p.Asp689Valfs∗2^e^ c.4485-21A > C,Cmp Het^e^		Cyriptic splice site Out-of-frame transcript truncated protein		Mild	Microcephaly ID, Rod-cone dysfunction	Mild
CM1 domain ɤ-TuRC targeting genes	CDK5RAP2	c.243T > A, p.Ser81X Hom^f^		Nonsense mutation	Truncated protein functional loss	Mild-moderate	Primary microcephaly (MCPH3) (Severe microcephaly) ID/MR	Simplified gyral pattern Reduced cerebral cortical volume Corpus callosum hypogenesis
		c.246T > A, p.Tyr82X Hom^g^		Nonsense mutation				
		c.IVS26-15A > G p.Glu385fs∗4, Hom^f^^,^^g^		Alternative splice acceptor site and premature termination codon				
		c.700G > T, p.Glu234X Hom^h^		Premature termination codon		Mild-moderate (+SNHL, hypotonia)		
		c.4546G > T, p.Glu1516X c.4672C > T, p.Arg1558X,Cmp Het^i^		Nonsense mutation		Severe (+mixed conductive-SNHL, simplified gyria, short stature		
		c.524_528del,p.Gln175Argfs∗42 c.4005-1G-A, Cmp Het^j^		Frameshift mutation, splicing defect, premature termination codon		Mild-moderate		
		c.4604+1G > C p.Val1526fs∗15 c.3097delG, p.Val1033fs∗41, Cmp Het^k^		Alternative splice acceptor site, premature termination codon, and frameshift mutation		Moderate (+cafe au lait lesions, facial dysmorphism)		
		c.4441C > T, p.Arg1481X Hom^l^		Nonsense mutation		Moderate (simplified gyria, CC agenesis)		

ɤ-TuSC, gamma-tubulin small complex; ɤ-TuRC, gamma-tubulin ring complex; TUBGCP-2,4,5,6, tubulin gamma complex associated protein-2,4,5,6; HAUS6, HAUS augmin-like complex subunit-6; NEDD1, neural precursor cell expressed developmentally down-regulated 1; CDK5RAP2, CDK5 regulatory subunit-associated protein 2; Hom, homozygous; Het, heterozygous; Cmp, compound; dup, duplication; del, deletion; Ala, alanine; Arg, arginine; Asp, aspartic acid; Cys: cysteine; Glu, glutamic acid; Gln, glutamine; Gly, glycine; Ile, isoleucine; Leu, leucine; Lys, lysine; Phe, phenylalanine; Pro, proline; Ser, serine; Trp, tryptophan; Tyr, tyrosine; Val, valine; DD, developmental delay; ID, intellectual delay; SNHL, sensory neural hearing loss; CC, corpus callosum; MCCRP, microcephaly and chorioretinopathy; MCPH3, primary microcephaly 3; MRI, magnetic resonance imaging.

a Mitani T, Punetha J, Akalin I, Pehlivan D, Dawidziuk M, Akdemir ZC, et al. Biallelic Pathogenic Variants in TUBGCP2 Cause Microcephaly and Lissencephaly Spectrum Disorders Am J Hum Genet. 2019:1–11. https://doi.org/10.1016/j.ajhg.2019.09.017.

b Scheidecker S, Etard C, Haren L, Stoetzel C, Hull S, Passemard S, et al. Mutations in TUBGCP4 Alter Microtubule Organization via the g -Tubulin Ring Complex in Autosomal-Recessive Microcephaly with Chorioretinopathy Am J Hum Genet. 2015 Apr 2; 96(4):666-74. https://doi.org/10.1016/j.ajhg.2015.02.011.

c Da Palma MM, Motta FL, Takitani GEDS, Salles MV, Lima LH, Ferraz Sallum JM. TUBGCP4 – associated microcephaly and chorioretinopathy Ophthalmic Genet. 2020 Apr; 41(2):189-193. https://doi.org/10.1080/13816810.2020.1747084.

d Maver A, Čuturilo G, Kovanda A, Miletić A, Peterlin B. Rare missense TUBGCP5 gene variant in a patient with primary microcephaly Eur J Med Genet. 2019 Dec; 62(12):103598. https://doi.org/10.1016/j.ejmg.2018.12.003.

e Hull S, Arno G, Ostergaard PIA, Pontikos N, Robson AG, Webster AR, et al. Clinical and Molecular Characterization of Familial Exudative Vitreoretinopathy Associated With Microcephaly Am J Ophthalmol. 2019 Nov; 207:87-98. https://doi.org/10.1016/j.ajo.2019.05.001.

f Bond J, Roberts E, Springell K, Lizarraga S, Scott S, Higgins J, et al. A centrosomal mechanism involving CDK5RAP2 and CENPJ controls brain size Nat Genet. 2005 Apr; 37(4):353-5. https://doi.org/10.1038/ng1539.

g Hassan MJ, Khurshid M, Azeem Z, John P, Ali G, Chishti MS, et al. Previously described sequence variant in CDK5RAP2 gene in a Pakistani family with autosomal recessive primary microcephaly BMC Med Genet. 2007 Sep 1; 8:58. https://doi.org/10.1186/1471-2350-8-58.

h Pagnamenta AT, Murray JE, Yoon G, Akha ES, Harrison V, Bicknell LS, et al. A Novel Nonsense CDK5RAP2 Mutation in a Somali Child With Primary Microcephaly and Sensorineural Hearing Loss Am J Med Genet A. 2012 Oct; 158A(10):2577-82. https://doi.org/10.1002/ajmg.a.35558.

i Lancaster MA, Renner M, Martin C, Wenzel D, Bicknell LS, Hurles ME, et al. Cerebral organoids model human brain development and microcephaly. Nature 2013; 501:373–9. https://doi.org/10.1038/nature12517.

j Tan CA, Topper S, Ward C, Stein J, Reeder A, Arndt K, et al. The first case of CDK5RAP2 -related primary microcephaly in a non-consanguineous patient identified by next generation sequencing. Brain Dev 2014; 36:351–5. https://doi.org/10.1016/j.braindev.2013.05.001.

k Pagnamenta AT, Howard MF, Knight SJL, Keays DA, Quaghebeur G, Taylor JC, et al. Activation of an exonic splice-donor site in exon 30 of CDK5RAP2 in a patient with severe microcephaly and pigmentary abnormalities Clin Case Rep. 2016 Aug 23; 4(10):952-956. https://doi.org/10.1002/ccr3.663.

l Issa L, Mueller K, Seufert K, Kraemer N, Rosenkotter H, Ninnemann O, et al. Clinical and cellular features in patients with primary autosomal recessive microcephaly and a novel CDK5RAP2 mutation. Orphanet J Rare Dis. 2013 Apr 15; 8:59. https://doi.org/10.1186/1750-1172-8-59).

Mutations in several components of the γ-tubulin complex including *TUBGCP4*, *TUBGCP5*, and *TUBGCP6* have been previously reported in human neurodevelopmental diseases often associated with microcephaly (Maver et al., 2019; Mitani et al., 2019; Scheidecker et al., 2015) (Da Palma et al., 2020; Hull et al., 2019; Maver et al., 2019; Mitani et al., 2019). Most of these mutations led to a loss of function and reduced levels of several GCP proteins (Table 1). Autosomal recessive variants in *TUBGCP2* encoding the γ-tubulin complex 2 (GCP2) protein were first reported in 5 individuals from 4 families with developmental delay, dysmorphic features, hypotonia, epilepsy, microcephaly, and lissencephaly spectrum changes on brain magnetic resonance imaging (pachygyria, agyria, subcortical band heterotopia), representing defective neuronal migration (Mitani et al., 2019). Thin corpus callosum, cerebellar and pons atrophy, and white matter abnormalities were also reported in some cases (Table 2). The authors speculated that the clinical phenotype was possibly due to a disturbed binding of different proteins to γ-tubulin or altered interactions between γ-tubulin complex proteins. However, no supporting functional data were provided that could shed light on the impact of these disease-causing variants on the mutant protein.

**Table 2:** Summary of the clinical presentation of patients with *TUBGCP2* mutations.

Case Gender Age	Origin/consanguinity/gestation	Lissence phaly	Microcephaly	Develop mental delay	Seizure-epilepsy onset/type	Other clinical features	Neurological examination	Physcomotor involvement	Brain MRI	Variant nucleotide/protein
Patient 1 Female 10 yo This paper	Turkish Yes Term	+	++	+	Intractable epilepsy 6 mo	Narrow forehead, thick eyebrows, prominent ear, bulbous nose, separated teeth, retrognathia	Hypotonia, muscle atrophy, contractures, spasticity, brisk DTRs	Loss of all motor and cognitive skills	10 y: Pachygyria, cerebral and cerebellar atrophy, cystic foci in white matter, and thinning of the corpus callosum	c.1015G > A p.Glu311Lys Homozygous
Patient 2 Male 6 yo This paper	Turkish Yes Term	+	++	+	Intractable epilepsy 3 yo	NA	Contractures, spasticity, increased DTRs	Prominent at 2 yo/walks with assistance	6 y: Pachygyria, cerebral and cerebellar atrophy, decreased white matter volumes, cystic foci at the centrum semiovale and thin corpus callosum	c.1015G > A p.Glu311Lys Homozygous
Family 1 Case 1 Male 6 yo [1]	Turkish Yes Term	+	+	+	Generalized seizures 6 y 9 mo	Narrow forehead, upslanting palpebral fissures, bulbous nose, prominent ear, widely spaced teeth, thick eyebrows, smooth philtrum, thin upper lip, pectus excavatum	Truncal hypotonia, normal DTRs, myopia	Delayed motor and language skills, autistic features	21 m: Pachygyria, thin corpus callosum, especially in the posterior region, mild cerebellar atrophy	c.997C > T p.Arg333Cys Homozygous *de novo* 2q23.1 dup (MBD5)
Family 1 Case 2 Male 7 yo [1]	Turkish Yes Term	+	+	+	No seizure	Narrow forehead, bulbous nose, prominent ear, smooth philtrum, retrognathia	Normal tone, normal DTRs	Normal motor skills, difficulty in reading	6 m: Posterior dominant pachygyria	c.997C > T p.Arg333Cys Homozygous
Family 2 Female 1yo 3mo [1]	Indian No Preterm (31 weeks)	+	+	+	Generalized seizures 5mo	Short and sloped forehead, thick eyebrows, puffy eyelids, full lips, retromicrognathia Exitus at ≅3 yo	Truncal hypotonia, brisk DTRs, spasticity, cortical blindness	Severely delayed motor and language skills	5 m: Pachygyria loss of white matter, thinning of the corpus callosum, volume loss of pons, and exuberant subependymal cyst formation, subependymal heterotipia, subcortical band	c.1843G > C p.Ala615Pro Homozygous
Family 3 Male 4 yo [1]	Iranian Yes Preterm (27 weeks)	+	+	+	Generalized seizures 7 mo	Bitemporal narrowing, upslanting palpebral fissure, micrognathia, midface hypoplasia, prominent ears and lips	Truncal hypotonia, no spasticity, optic atrophy, retinal changes	Severely delayed motor and language skills	1 year: Pachygyria, hyperintense periventricular white matter, very thin corpus callosum, and subependymal cysts, subcortical band, thin pons	c.1843G > C p.Ala615Pro Homozygous
Family 4 Male 8 yo [1]	Polish No Term	+	+	+	Generalized seizures	Smooth philtrum, prominent ears	Normal DTRs, no spasticity, myopia, astigmatism	Delayed motor and language skills	8 y: Pachygyria in the temporal lobes and partial thinning of the corpus callosum	c.889C > T p.Arg297Cys c.2025-2A > G Com Het

MRI, magnetic resonance imaging; yo, years old; mo, months; DTR, deep tendon reflexes.

MTs are one of the main cytoskeleton builders and are involved in many important functions such as intracellular transport, organelle positioning, motility, signaling, and cell division (Brouhard and Rice, 2018; Vale, 2003). MTs are long fibers of 25 nm in diameter made of 13 polarized protofilaments in mammals, each protofilament composed of α- and β-tubulin heterodimers (de Pablo et al., 2003). The polarity of the tube provides specific dynamic characteristics to the ends where different polymerization and depolymerization reactions occur (Brouhard and Rice, 2018). MTs are mainly formed at the MT organizing centers (MTOCs), the centrosome being the most important MTOC in mammals (Wu and Akhmanova, 2017). Centrosomes are organelles composed of two perpendicular barrels of 9 triplets of MTs surrounded by a proteinaceus matrix called the pericentriolar material (PCM) (Fry et al., 2017). Cryo-electron microscopy studies on structure of the human γTuRC, combined with cross-linking mass spectrometry analysis, reveal an asymmetric conformation with only part of the complex in a “closed” conformation, while the opposite side of γTuRC is in an “open” conformation, leading to a structural asymmetry suggesting possible regulatory mechanisms for MT nucleation by γTuRC closure (Consolati et al., 2020; Rale et al., 2018). This complex named γ-tubulin ring complex or γ-TuRC was found to work as an MT nucleation complex (Tovey and Conduit, 2018). Further biochemical analysis identified at least seven proteins co-purifying with γ-tubulin in mammalian cells, known as γ-tubulin complex proteins or GCPs (GCP2-6) (Yu et al., 2016). One molecule of GCP2 together with one molecule of GCP3 and two molecules of γ-tubulin form a γ-tubulin small complex or γ-TuSC, the basic unit of the γ-TuRC (Raynaud-Messina and Merdes, 2007). A full γ-TuRC consists of several γ-TuSC associated with a few additional GCPs. In addition to its nucleating activity, the γ-TuRC also acts as a minus-end capping complex, therefore stabilizing MTs.

The γ-TuRC is targeted to the centrosome through the neural precursor cell expressed developmentally down-regulated protein 1 (NEDD1). The N-terminal part of NEDD1 contains a conserved WD40 domain necessary for centrosome binding, while the C-terminal part is required for γ-tubulin interaction (Yonezawa et al., 2015). Different phosphorylations in NEDD1 control not only the targeting of γ-TuRC to the centrosome but also the spatial and temporal regulation of MT nucleation at different sites in the cell (Gomez-Ferreria et al., 2012). For instance, a recently described mechanism explains acentrosomal MT assembly in mitosis by an octameric complex of proteins termed the Augmin complex. This eight-subunit complex is conserved in animal and plants and is composed of the HAUS proteins (HAUS1-8). HAUS6 binds to γ-TuRC while HAUS8 directly binds to the lattice of a pre-existing MT, creating an MT nucleation point and, thus, an MT branching point (Lawo et al., 2009).

In this report, we studied the localization of several components of the γ-TuRC complex in control and *TUBGCP2* mutated human fibroblasts, as well as the levels of the TUBGCP2 protein along the cell cycle, and performed proteomics and structural modeling studies to explore the functional effect of the mutant TUBGCP2 protein in neurodegeneration.

## Results

### Patients

We studied 2 siblings born to consanguineous Turkish parents. Patients and family members were recruited at the Department of Paediatric Neurology, Malatya (Turkey) after informed consent. Samples were pseudo-anonymized, processed, and stored within the MRC Centre for Neuromuscular Diseases Biobank (National Research Ethics Service, Newcastle and North Tyneside 1 Research Ethics Committee: REC reference number 08/H0906/28 + 5).

**Figure 1:**
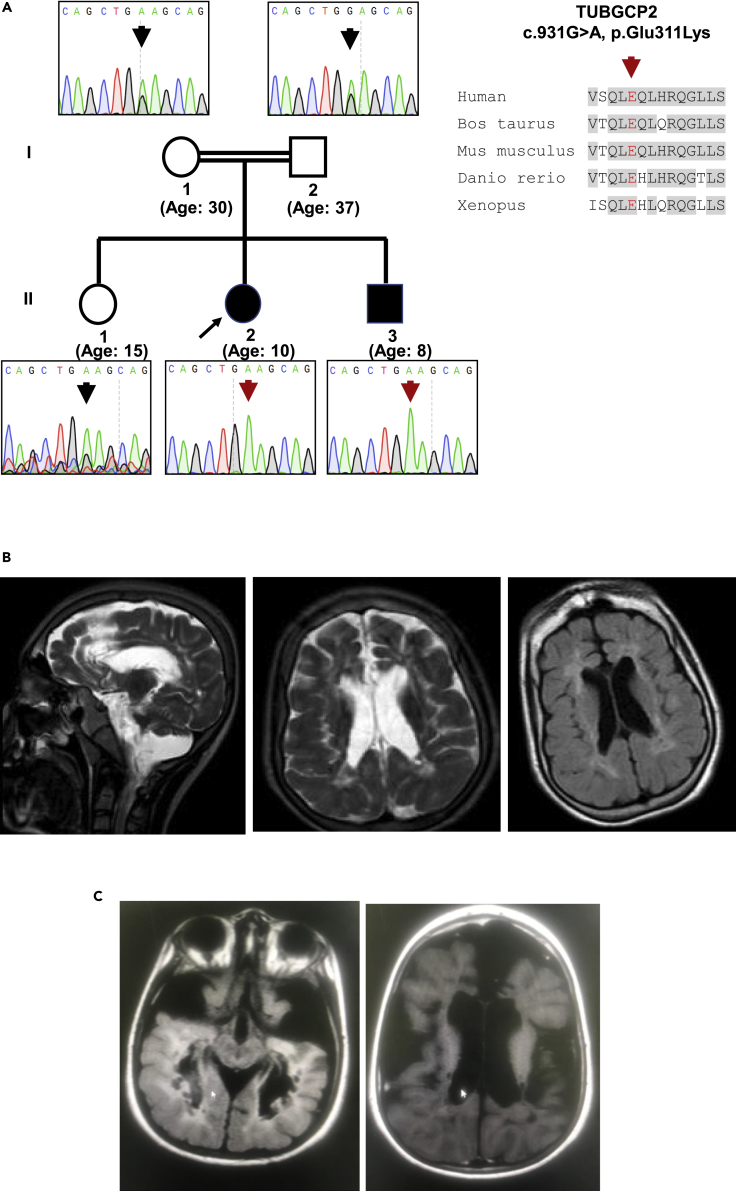
Clinical presentation and brain MRI of the patients (A) Pedigree and sequence data including the conservation of the protein. (B) Cranial T2-weighted MR images of the index patient showed pachygyria, cerebellar parenchymal atrophy, bilateral volume loss in cerebral white matter, cystic foci with increased intensity in the neighboring white matter, and thinning of the corpus callosum. (C) Her affected sibling's MRI detected cerebral and cerebellar atrophy and cystic foci with decreased white matter volumes.

The 10-year-old female patient was the second child born to first cousin parents (Figure 1A). She presented with severe developmental delay, hypotonia, and intractable epilepsy at age 6 months and lost all motor and cognitive abilities gradually by 4 years of age. She presented dysmorphic features including narrow forehead, thick eyebrows, bulbous nose, prominent ear, widely separated teeth, retrognathia, and maxillary hypoplasia (Table 2, Figure 1B). Neurological examination revealed microcephaly, atrophy, and contractures of the extremities with brisk deep tendon reflexes and spasticity. Cranial T2-weighted magnetic resonance (MR) images showed pachygyria, cerebellar parenchymal atrophy, bilateral volume loss in cerebral white matter, cystic foci with increased intensity in the neighboring white matter, and thinning of the corpus callosum (Figure 1B). Her 8-year-old brother demonstrated normal developmental milestones until 8 months of age, when developmental delay was noticed and became evident after 2 years of age. Intractable seizures started after 3 years of age. His neurological examination at 8 years of age revealed microcephaly, atrophy and contractures of the extremities, increased deep tendon reflexes, and spasticity. He was able to walk with assistance. Cranial MR detected cerebral and cerebellar parenchymal atrophy, significantly decreased white matter volumes, cystic foci with neighboring hyperintensities at the centrum semiovale and thin corpus callosum (Figure 1C and Table 2).

### Genetic analysis by whole exome sequencing

We performed whole exome sequencing (WES) in both siblings and their parents as described previously (Balaraju et al., 2020). We identified a homozygous variant in *TUBGCP2* exon 8 *(*NP_006650.1: c.931G > A, p.Glu311Lys, hg19 chr10:135106636) within the extended Grip1 domain in both siblings, while both parents and a 15-year-old healthy sibling are heterozygous carriers (Figure 1A). This variant has not been reported previously and not present in gnomAD or in a cohort of 1,182 ethnically matched Turkish control individuals (TUBITAK MAM-GMBE data set: http://gmbe.mam.tubitak.gov.tr/en). *In silico* analysis suggested that c.931G > A, p.Glu311Lys is deleterious, using prediction tools such as Polyphen2 (http://genetics.bwh.harvard.edu/pph2/), CADD (https://cadd.gs.washington.edu/), and SIFT (http://sift.jcvi.org/) to assess pathogenicity. Sanger sequencing confirmed that this variant is homozygous in the patients and heterozygous in the healthy parents.

### Analysis of components of γ-TuRC

**Figure 2:**
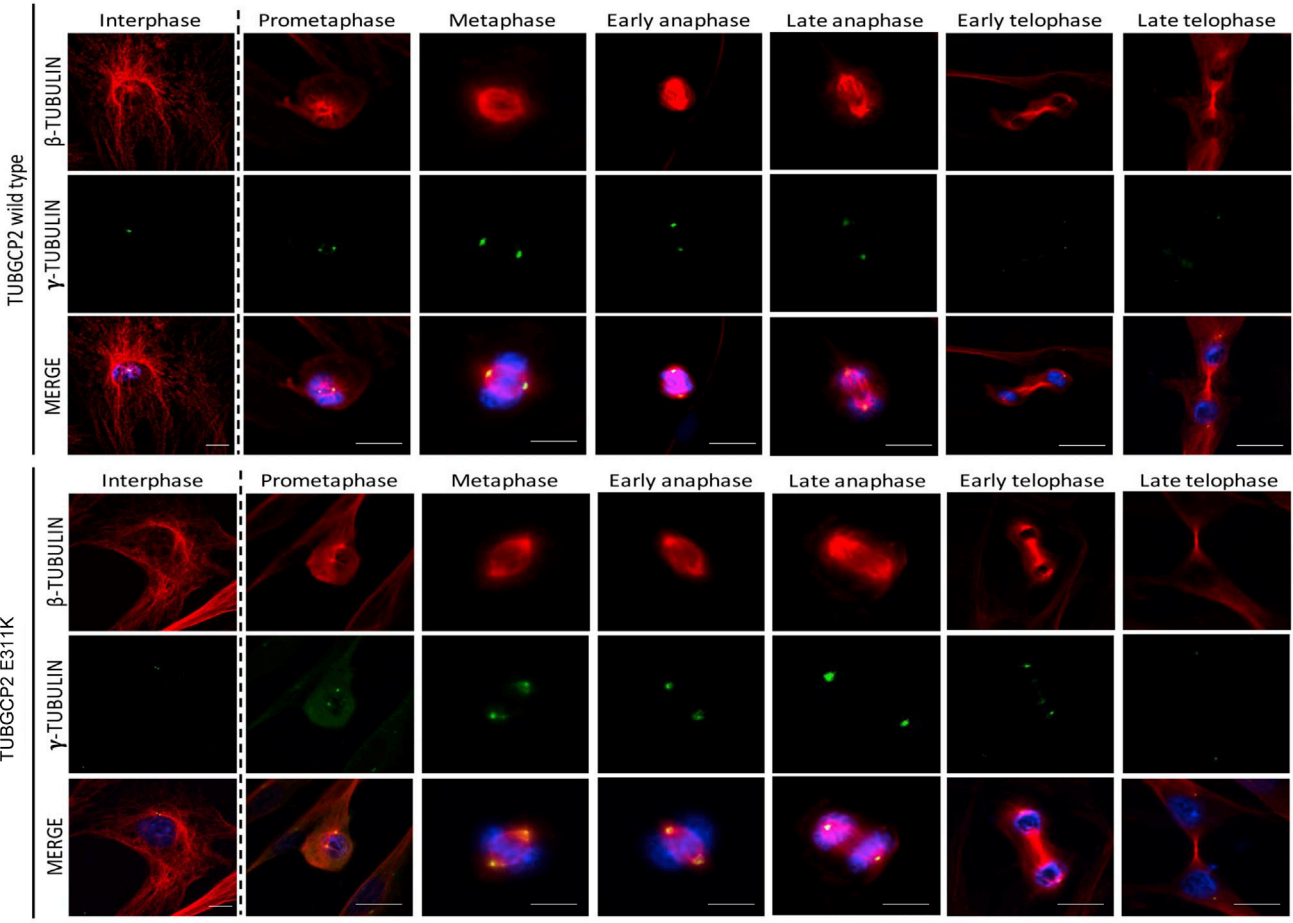
γ-Tubulin localization is affected in TUBGCP2 p.Glu311Lys (E311K) fibroblasts Asynchronous cells were stained for γ-tubulin (green), β-tubulin (red), and DNA (blue), and different phases of mitosis were captured. Scale bar, 10 μm.

**Figure 3:**
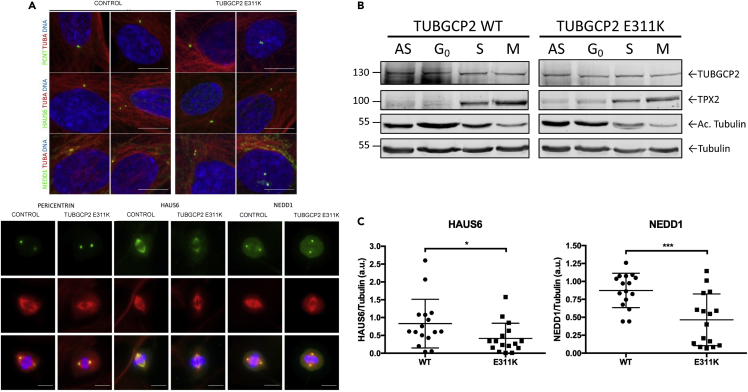
Similar TUBGCP2 levels are present in control and patient fibroblasts along the cell cycle (A) Asynchronous cells were stained for either PCNT, HAUS6 or NEDD1 (green), ⍺- or β-tubulin (red), and DNA (blue), and interphase cells (upper panel) or different phases of mitosis (lower panel) were captured. Immunofluorescence images of wild-type and *TUBGCP2* mutant (p.Glu311Lys) fibroblasts in metaphase showing that the localizations of HAUS6 and NEDD1 are altered in the mutant cells. Scale bar, 10 μm. (B) Similar TUBGCP2 levels are present in control and patient fibroblasts along the cell cycle. Cells were synchronized in G_0_ (48 hr of serum starvation), S phase (double thymidine block), and mitosis (double thymidine/nocodazole block), and 50 μg of total cell lysate was loaded onto 10% SDS-PAGE. Antibodies used in this Western blot were as follows: rabbit a-TPX2 (1 ug/ml), rabbit anti-TUBGCP2 (1:2000), mouse anti-AcTubulin (1:1000), and rabbit anti-tubulin (1:500). Scale bar, 10 μm. (C) The signal intensity of HAUS6 and NEDD1 was quantified and normalized to either the α- or β-tubulin signal intensity depending on the combination of antibodies used. Fifteen metaphases have been analyzed for each condition and represented in scatted plots. Data are represented as mean ± SEM. ∗P < 0,05 and ∗∗∗P < 0.001, Student's t-test).

As TUBGCP2 is a core component of the γ-TuRC nucleation complex, we studied the localization of some γ-TuRC components and associated proteins in control and *TUBGCP2* mutated human fibroblasts in interphase and in mitosis by immunofluorescence, as well as the levels of the GCP2 protein along the cell cycle (Figure 2). We observed a faint delocalization of γ-tubulin in the mitotic cells of the patient fibroblasts. This suggested that the mutation in TUBGCP2 could perturb γ-TuRC localization pattern. To test this hypothesis, we looked at the localization of other components of the γ-TuRC complex such as HAUS augmin-like complex subunit 6 (HAUS6), protein NEDD1 (NEDD1), and pericentrin (PCNT), an integral component of the PCM of the centrosome involved in the initial establishment of organized MT arrays in mitosis (Figure 3A). We did not detect any significant changes in the patient fibroblasts in interphase (Figure 3A upper panel). However, in mitosis, patient fibroblasts presented a faint delocalization of two components associated with the γ-TuRC complex, HAUS6 and NEDD1 (Figure 3A, lower panel, and 3C). The localization of HAUS6 was clearly affected at all stages of mitosis as the protein presents with a diffuse pattern throughout the cytoplasm (Figure 3A). In contrast, there was no visible effect on the centrisomal localization of PCNT neither in interphase nor in mitosis.

Next, we wondered whether the levels of TUBGCP2 in the patient fibroblast could be altered and, in turn, affect the localization of other γ-TuRC components or associated proteins. To test this hypothesis, we synchronized control and patient fibroblasts and checked the levels of TUBGCP2 (Figure 3B). As synchronization and loading controls, we used acetylated tubulin (increased in G_0_) and TPX2 (increased in mitosis). We did not observe any significant change in the levels of TUBGCP2 in control and patient fibroblasts along the cell cycle. Acetylated tubulin was increased in G_0_ and TPX2 in mitosis, confirming a correct synchronization of cells.

### Structural modeling of the *TUPBGCP2* missense mutation

**Figure 4:**
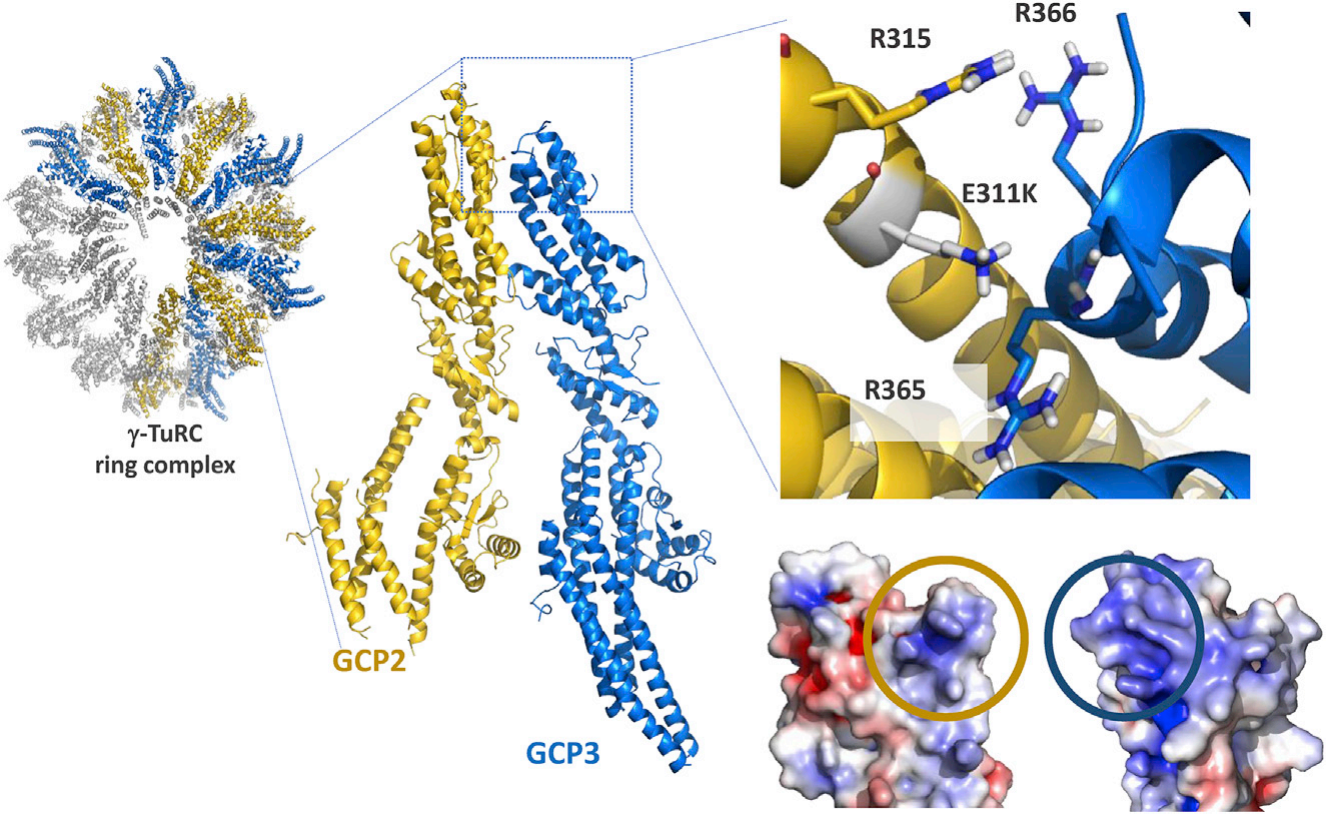
Computational modeling of the E311K mutation The γ-TuRC ring complex contains five repeating units of GCP2 (gold cartoon) and GCP3 (marine cartoon) complex (pdb id: 6v6s). The acidic GCP2-E311 is complemented by the basic environment made of three arginine residues (R315 of GCP2, R365, and R366 of GPC3). The indicated charge complementarity will be lost upon E311K mutation. This is depicted by the mutation-induced change in the electrostatic distribution of GCP2, facing GCP3. The interacting surfaces of GCP2 and GCP3 are encircled in gold and marine, respectively.

GCP2:CGP3 inter-molecular interactions make up nearly half of the γ-TuRC ring complex (Wieczorek et al., 2020) (Figure 3C). The E311 of GCP2 is located across the interaction core of each asymmetric GCP2:GCP3 complex with 3,000 A ° 2 interface between GCP2 and GCP3 within the complex. The acidic nature of E311 is complemented by the surrounding basic residues of GCP2 (R315) and GCP3 (R365 and R366 of GCP3). The E311K mutation of GCP2 induces a disruption in this complementarity. This is highlighted with the mutation-induced change in the electrostatic surface of GCP2, facing to GCP3 (Figure 4). As a result, the rather acidic GCP2 patch gets modified into a basic one, which would be repelled by the basic GCP3 surface.

### Proteomics studies with label-free liquid chromatography mass-spectrometry

We applied proteomics to study the functional effect of the *TUBGCP2* mutation in fibroblasts. Proteomics allows the unbiased discovery of pathophysiological processes in rare neurodegenerative and neuromuscular diseases (Roos et al., 2018), and based on previous studies, fibroblasts were proven to represent a suitable model to study the molecular etiology of neurological diseases (Mingirulli et al., 2020) (Hentschel et al., submitted to this issue). Therefore, we analyzed a human skin fibroblast protein library for the expression of TUBGCP2 and identified 14 unique peptides covering 23% of the entire protein (Figure S2). This result demonstrates the expression of TUBGCP2 in human fibroblasts and thus indicates the suitability of these cells to study the effect of TUBGCP2 mutations *in vitro*. Moreover, expression data of TUBGCP2 (https://gtexportal.org/home/gene/TUBGCP2) show that this protein is highly expressed in fibroblasts and skin, in similar levels with the brain cerebellum which has one of the highest expression levels of *TUBGCP2* (Figure S1) reinforcing the suitability of this cellular model.

**Figure 5:**
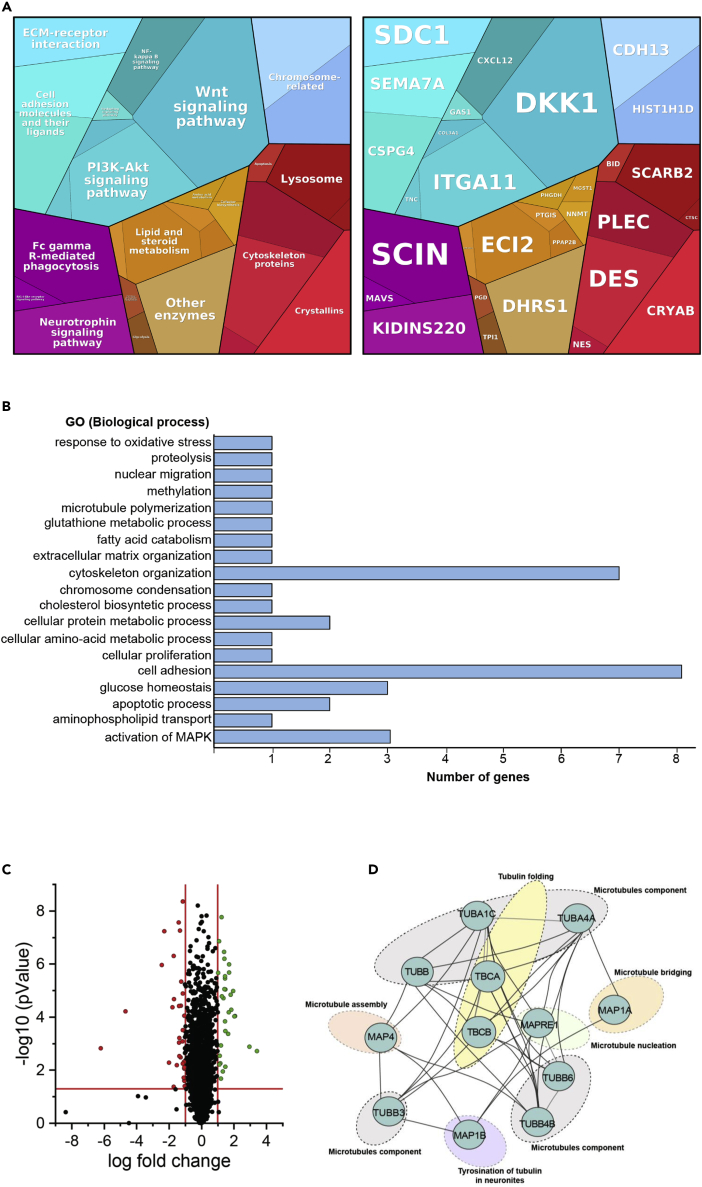
Proteomics analysis of TUBGCP2 and control fibroblasts (A) Proteomap resulting from the comparative proteome profiling of TUBGCP2 fibroblasts versus control cells. Every polygon or circle represents a protein, the size of which is given by the fold change. The proteins are then grouped in functional categories based on the KEGG database. The proteomap shows five main hierarchy levels, which are further divided into sub-pathways. (B) In silico analysis of dysregulated proteins utilizing GO term (biological pathway) annotation showing that proteins involved in cell adhesion and cytoskeleton organization are majorly affected by the TUBGCP mutation. (C) Volcano plot shows proteins which are significantly increased or decreased, compared to control fibroblasts. (D) String analysis visualized using Cytoscape of microtubule and microtubule-associated proteins identified within our proteomic analysis.

Next, we applied a label-free liquid chromatography mass-spectrometry (LC-MS/MS) approach to investigate the proteomic signature of human skin fibroblasts derived from the index patient with the homozygous *TUBGCP2* c.931G > A, p.Glu311Lys mutation. This unbiased study revealed a statistically significant (p-ANOVA ≤ 0.05) dysregulation of 50 proteins: 26 were increased and 24 decreased (Table S1; ≤0.46 = significantly decreased and ≥2.24 = significantly increased). Further *in silico-*based pathway analyses (proteomaps based on the “Kyoto Encyclopedia of Genes and Genomes” [KEGG]; [Liebermeister et al., 2014]) of these proteins suggested that proteins involved in the assembly and organization of the cytoskeleton and the extracellular matrix are affected along with proteins controlling cellular adhesion. In addition, our proteomic findings raise the possibility that *TUBGCP2* mutations affect other cellular processes such as different metabolic (glycolysis, lipid and sterol oxidation, and amino acid metabolism) and signaling (MAPK, PI3K-AKT, and WNT) pathways (Figure 5A). Results of a gene ontology-based analysis of our proteomic data revealed that proteins crucial for neuronal homeostasis including axon guidance are also affected (Figures 5B and 5C and Table S1). Further analysis of functional protein association networks via STRING (and Cytoscape [Shannon et al., 2003]) indicated a potential functional interplay of several proteins affected by mutant TUBGCP2 (Figure 5D). In addition, we analyzed the abundance of 8 tubulins identified in our comparative proteomic profiling approach regardless of the above mentioned cut-off values for up- or down-regulation. One (tubulin beta-3 chain) shows an increase of more than 25%, whereas three (tubulin beta chain, tubulin beta-4B chain, and tubulin alpha-1C chain) presented with more than 25% decrease in abundance (Figure 5D) indicating an effect of *TUBGCP2* mutations on other tubulin proteins.

### Immunofluorescence studies on human skin fibroblasts confirm proteomic findings

**Figure 6:**
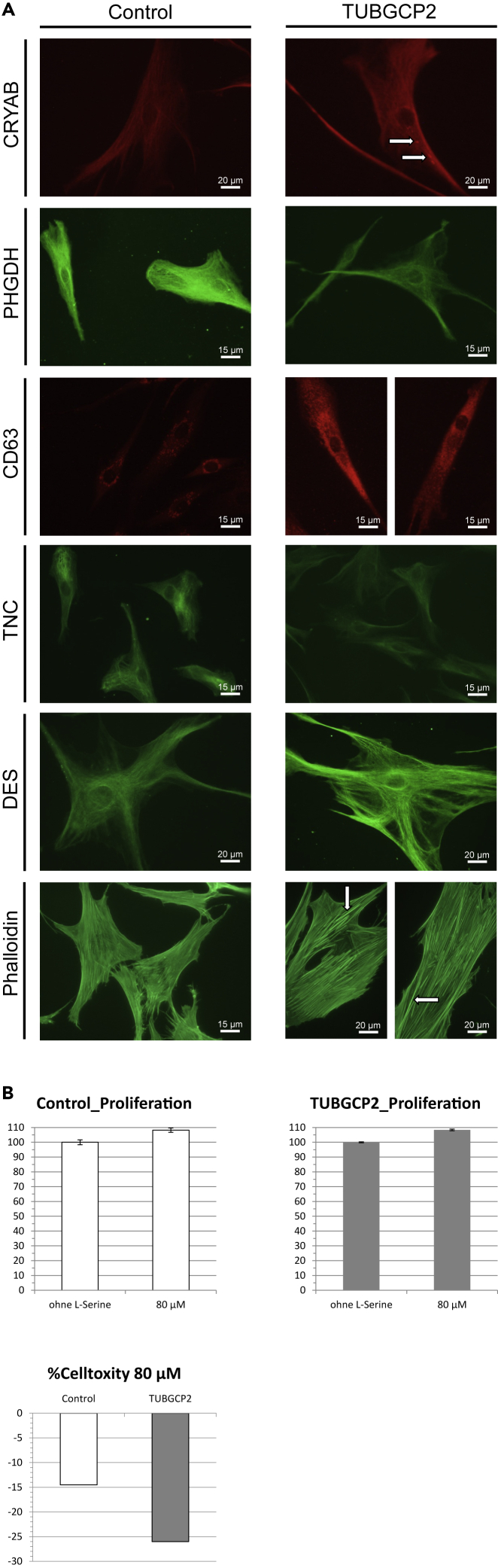
Immunohistochemical studies confirmed proteomics findings in patient fibroblasts (A) Immunofluorescence studies on TUBGCP2-patient-derived fibroblasts detected increased abundance of αB-crystallin (CRYAB) (white arrows), CD63, desmin, and phalloidin, while reduced levels of D-3-phosphoglycerate dehydrogenase (PHGDH) and tenascin (TNC), confirming the findings detected by proteomics analysis. Scale bar is shown on each image. (B) L-serine treatment in cultured skin fibroblasts revealed an 8% increased proliferation in both TUBGCP2-patient and control, while a 26% reduction of cytotoxicity was detected in patient-derived cells compared to 14% in controls. These changes did not reach statistical significance. Data are represented as mean ± SEM.

Immunofluorescence studies on TUBGCP2-patient-derived and control fibroblasts were carried out to validate our proteomic findings. In line with our mass spectrometric based protein quantification, immunological investigation of αB-crystallin (CRYAB) revealed increased abundance with focal cytoplasmic accumulations (white arrows) in patient-derived cells (Figure 6A). Studies of D-3-phosphoglycerate dehydrogenase (PHGDH) and tenascin confirmed the reduced abundances of both proteins as identified by proteomic profiling (Figure 6A). Prompted by the identified increase of lysosome membrane protein 2 (Figure 5) indicative for increased activation of a lysosomal protein degradation pathway, we investigated levels of CD63, a member of the tetraspanin superfamily commonly used as a marker of late endosomes and lysosome-related organelles. Compared to control cells, fibroblasts derived from the TUBGCP2-patient presented with a profound increase of CD63 immunoreactivity (Figure 6A). In accordance with our proteomic findings, immunofluorescence studies of desmin (DES) revealed an increased level of this type III intermediate filament in the TUBGCP2-patient-derived fibroblasts (Figure 6A). Prompted by the general vulnerability of cytoskeletal and cytoskeleton remodeling proteins in patient-derived cells including increase of adseverin, a Ca^2+^-dependent actin filament-severing protein, we investigated F-actin level and distribution by FITC-phalloidin staining. Results of these studies revealed increase of thicker actin bundles (Figure 6A) most likely referring to actin stress fibers in patient cells.

### L-serine supplementation reduces cytotoxicity in TUBGCP2-patient-derived fibroblasts

Proteomic profiling identified PHGDH as a protein significantly altered in abundance in the *in vitro* model of TUBGCP2. Given that recessive PHGDH mutations also result in a neurological phenotype and that PHGDH-patients respond to L-serine treatment, the effect of L-serine treatment was pre-clinically addressed in cultured skin fibroblasts derived from the TUBGCP2-patient: although investigation of the proliferation revealed an increase of 8% in fibroblasts of both, TUBGCP2-patient and control upon L-serine supplementation, in patient-derived cells, a 26% reduction of cytotoxicity was detected compared to 14% in control cells (Figure 6B).

## Discussion

MTs are long fibers made of 13 protofilaments of α- and β-tubulin heterodimers (Wu and Akhmanova, 2017). Considered as one of the main cytoskeleton elements, they are involved in intracellular transport, organelle positioning, motility, signaling, and cell division (Kollman et al., 2010). MTs are nucleated at the MT organizing centers, most importantly the centrosome, which is an organelle composed of 2 perpendicular barrels of 9 triplets of MTs surrounded by the pericentriolar material (Teixido-Travesa et al., 2012). The levels of TUBGCP2 were comparable in control and patient fibroblasts suggesting that the stability of the mutated protein along the cell cycle is not affected. In fibroblasts of the patient, the protein steady state level of TUBGCP2 was not significantly altered. However, in mitosis, patient fibroblasts presented a faint delocalization of two components associated with the γ-TuRC complex (HAUS6 and NEDD1, Figure 3). Expanding on our structural analysis, we propose that this mutation impacts the stability of the γ-TuRC ring potentially caused by the charge swap introduced by the c.931G > A, p.Glu311Lys variant, which is predicted to change the electrostatic complementarity of the GCP2:GCP3 interface. The c.931G > A, p.Glu311Lys mutation seen in our patient is situated between the two Grip1 domain variants previously reported, namely c.889C > T (p.Arg297Cys) and c.997C > T (p.Arg333Cys). Our data suggest that in contrast to as hypothesized by Mitani et al. (Mitani et al., 2019), mutations in the Grip1 domain may affect the localization of γ-TuRC and the mutation does not impinge on the steady-state level of the GCP2 protein (Figure 3).

Results of our proteomic profiling revealed the altered abundance of a total of 50 proteins suggesting a cellular vulnerability against homozygous *TUBGCP2* missense mutations. Interestingly, other tubulin proteins are affected only to a minor degree (Figure 5). The cytoskeleton appears to be affected by the expression of mutant TUBGCP2, as several proteins crucial for the assembly and maintenance of cellular cytoskeleton such as DES, plectin, adseverin, PDZ and LIM domain protein 5, syndecan, nestin, and EH domain-binding protein 1 are dysregulated. Notably, some of those cytoskeletal proteins are known to be involved in neuronal functions: Nestin overexpression has been shown to be crucial for brain development by regulating cell proliferation and neuronal progenitor cell division; it is used as a marker of neuronal progenitor cells (Liu et al., 2015). Syndecan-1 regulates the maintenance and proliferation of neural progenitor cells during mammalian cortical development, which has potential relevance for the prominent neuronal migration defects seen in the patients (Wang et al., 2012).

Pathogenic amino acid substitutions in TUBGCP2 may also lead to dysregulation of proteins involved in cellular adhesion to the extracellular matric (ECM), an important process for cell migration and invasion. Both processes are tightly associated with the MT network (Seetharaman and Etienne-Manneville, 2019) (Figure 5). For example, integrin signaling plays a crucial role in cell adhesion by altering MT stabilization, organization, and dynamics. Of note, our data suggest altered expression of integrin alpha-11, semaphorin 7A (Pasterkamp et al., 2003), and matrix-remodeling-associated protein 8 (Jung et al., 2012) (Table S1) supporting the concept of a possible perturbed integrin signaling in TUBGCP2-patient-derived cells. Moreover, numerous studies of initial myelination and remyelination stages in the central nervous system demonstrated the importance of a functional interplay between several key cytoskeletal components and integrin superfamily proteins, which is in line with the white matter abnormalities detected in our patients (e.g. [Miyata, 2019]).

Interestingly, *TUBGCP2* mutations may also affect metabolic processes, some of which are of great importance in neuronal cells (Figure 5): PHGDH, the first step enzyme in the *de novo* production of L-serine, an amino acid crucial for brain development and neuron survival (Hirabayashi and Furuya, 2008) was found to be decreased in patient-derived *TUBGCP2*-mutant fibroblasts. Several publications highlighted the importance of L-serine in central nervous system (CNS) development and maintenance, and supplementation with L-serine was found to have a beneficial effect in motor neuron disease (Levine et al., 2017) linked to neuroprotection through the modulation of the endoplasmic reticulum (ER) stress response (Dunlop et al., 2018) and in hereditary sensory and autonomic neuropathy due to mutations in *SPTLC1* (Fridman et al., 2019).

Of note, PHGDH deficiency was linked to a neurological disease defined by congenital microcephaly, psychomotor retardation, and seizures, as well as neuropathy (Jaeken et al., 1996; Poli et al., 2017). Prompted by the neurological phenotype observed in our patients and the above mentioned impact of L-serine produced by PHGDH on neuronal function and survival, we preclinically tested the effect of L-serine supplementation on fitness of patient-derived fibroblasts. Results of these studies demonstrated a beneficial effect of L-serine treatment in fibroblasts, a valuable model to study the molecular etiology of neurological diseases (Hentschel et al., preprint available https://doi.org/10.21203/rs.3.rs-48014/v1), thus suggesting that L-serine treatment might represent a concept to ameliorate the phenotype.

Although MT polymerization has been claimed to have an impact on several metabolic processes, such as glycolysis (Cassimeris et al., 2012), we could observe indeed a decrease of proteins involved in glycolysis (triosephosphate isomerase), gluconeogenesis (6-phosphogluconate dehydrogenase), and glucose homeostasis (insulin-like growth factor-binding protein 5) in TUBGCP2-deficient fibroblasts. These processes are crucial for proper brain functioning, and their dysregulation has already been linked to the manifestation of neurological diseases (Mergenthaler et al., 2013).

Proteomic profiling also suggested that proteins involved in the activation of MAPK may be up-regulated in the patient-derived cells and might be involved in the molecular etiology of the disease: Kinase D-interacting substrate of 220 kDa is a multifunctional scaffold protein involved in neuronal development, neurite outgrowth, and maturation (Scholz-Starke and Cesca, 2016) and its increase in TUBGCP2-patient-derived fibroblasts might reflect a possible rescue mechanism. In contrast, an increase in chondroitin sulfate proteoglycan 4, as identified in patient-derived fibroblasts, may be associated with the inhibition of functional recovery by impeding axonal sprouting and synaptic rearrangements as suggested previously (Loers et al., 2019).

Several proteins dysregulated upon the homozygous *TUBGCP2* missense mutation play crucial roles in the development and maintenance of the nervous system, highlighting that axon and neurite outgrowth/elongation may be affected along with perturbed neuronal differentiation, migration, and synaptic plasticity. Hence, our proteomic findings obtained in primary patient fibroblasts hint toward possible pathophysiological downstream effects of *TUBGCP2* mutations on normal development and functioning of the nervous system and thus provide insight for the clinical manifestation of *TUBGCP2*-associated neuropediatric disease. Moreover, human skin fibroblasts show promise to further delineate the pathophysiology and explore potential treatments for this rare disorder.

In summary, we describe two siblings carrying a homozygous *TUBGCP2* variant with a severe phenotype, and show, that in addition to a neuronal migration defect, brainstem atrophy and disturbed myelination may also be associated with *TUBGCP2* mutations, explaining the variable clinical and imaging findings.

### Limitations of the study

In this study, we used human primary fibroblasts of a patient with pathogenic mutations to reveal molecular insights into the pathomechanism of a severe childhood-onset neurological disease. Fibroblasts may not be the best cell type representing neuronal cells. However, the mutant protein is expressed in fibroblasts, and we believe that our results provide relevant information on the effect of the mutant protein also in other cells types, such as the neurons, neural progenitor cells, etc. Using fibroblasts and not neuronal cells for functional studies may be a limitation of the model.

### Resource availability

#### Lead contact

The main point of contact for responding to material and resource requests is Dr. Rita Horvath (Department of Clinical Neurosciences, University of Cambridge).

We are happy to reply to requests regarding Materials, Data and Code in this publication.

#### Material availability

All the materials, data generated or analyzed during this study are included in this article or in the supplemental Transparent methods and are available from the corresponding author upon request.

#### Data and code availability

All genetic data have been deposited in the EGA database and in RD-CONNECT under the following ID numbers: patient 1: E497133, patient 2: E477343, mother: E615258, father: E739679, unaffected sibling: E191145. These data can be made available after an authentication process.

## Methods

All methods can be found in the accompanying Transparent Methods supplemental file.

## Supplemental information

Document S1. Transparent methods, figures S1and S2, and table S1
