# Supplementary material for: Autosomal recessive variants in TUBGCP2 alter the γ-tubulin ring complex leading to neurodevelopmental disease
Source: iScience. 2020 Dec 30;24(1):101948. doi: 10.1016/j.isci.2020.101948 (PMC7797523; doi:10.1016/j.isci.2020.101948)
Supplement: Document S1. Transparent methods, figures S1and S2, and table S1 [file mmc1.pdf]

iScience, Volume *24*

Supplemental Information

Autosomal recessive variants in *TUBGCP2* alter the γ-tubulin ring complex leading to neurodevelopmental disease

Serdal Gungor, Yavuz Oktay, Semra Hiz, Álvaro Aranguren-Ibáñez, Ipek Kalafatcilar, Ahmet Yaramis, Ezgi Karaca, Uluc Yis, Ece Sonmezler, Burcu Ekinci, Mahmut Aslan, Elmasnur Yilmaz, Bilge Özgör, Sunitha Balaraju, Nora Szabo, Steven Laurie, Sergi Beltran, Daniel G. MacArthur, Denisa Hathazi, Ana Töpf, Andreas Roos, Hanns Lochmuller, Isabelle Vernos, and Rita Horvath

Supplementary information

Autosomal recessive variants in *TUBGCP2* alter the γ-tubulin ring complex leading to neurodevelopmental disease

Transparent methods

Whole exome sequencing (WES) and bioinformatics analysis

Patients and family members were recruited at the Department of Paediatric Neurology, Malatya (Turkey) after informed consent. Samples were pseudo-anonymized, processed and stored within the MRC Centre for Neuromuscular Diseases Biobank (National Research Ethics Service, Newcastle and North Tyneside 1 Research Ethics Committee: REC reference number 08/H0906/28+5).

WES of the female patient, parents and the affected brother was performed by the Genomics Platform at the Broad Institute of MIT and Harvard, Cambridge, USA (Yaramis et al., 2020). Libraries were created with an Illumina exome capture (38 Mb target) and sequenced with a mean target coverage of >80x. Genomic and phenotypic data were submitted to the RD-Connect Genome-Phenome Analysis Platform, GPAP, (https://platform.rd-connect.eu), where they can be accessed under a controlled access agreement. Exome sequencing data were processed and analysed on the RD-Connect GPAP. Likely pathogenic variants were identified applying standard filtering for high to moderate variant effect predictor (i.e. nonsense, splice site, frame-shift, in-frame and non-synonymous variants), and for minor allele frequency <1% in gnomAD (http://gnomad.broadinstitute.org), and in a cohort of 1,182 ethnically-matched Turkish control individuals (TUBITAK MAM-GMBE dataset: http://gmbe.mam.tubitak.gov.tr/en). Shortlisted variants were interrogated for their predicted *in silico* deleteriousness, previous known association with human disease and were

classified by the ACMG Guidelines. Likely pathogenic variants were segregated in unaffected siblings by Sanger sequencing. All exome data are available in the RD-CONNECT platform.

Analysis of components of γ-TuRC

Immunofluorescence images were taken of human fibroblasts grown on coverslips and MetOH fixed. Permeabilization and blocking of fibroblasts was carried out in IF buffer (0,5% BSA, 0,1% Triton X-100 in PBS) for 30 min at RT. After blocking and permeabilization, human fibroblasts were incubated with primary antibodies diluted in IF buffer for 1 h at RT and then 3 washes of 5 min in IF buffer were performed. Samples were then incubated with secondary antibodies for 45 min at RT and washed once with IF buffer and twice with PBS. Antibodies and concentrations were the following: mouse anti-γ-tubulin (1:1000, Sigma, T6199), rabbit anti-PCTN (1:500, Abcam, Ab4448), rabbit anti-HAUS6 (1:2000, Homemade), anti-NEDD1 (1:1500, Abnova, H00121441-M05), mouse anti-α-tubulin (1:1000, Sigma, T9026), rabbit anti-β-tubulin (1:350, Abcam, Ab6046).

For western blot analysis, human fibroblasts were synchronized with a double thymidine (2 mM, overnight) nocodazole (0,33 uM, overnight) block and then lysed in lysis buffer (50 mM Tris-HCl pH 7,4, 150 mM NaCl, 1% NP-40, 1 mM EDTA and protease inhibitors). 30 ug of total cell lysates were subjected to 8% SDS-PAGE and blotted onto nitrocellulose membranes (Millipore). Membranes were blocked with 3% BSA in TBS-Tween 0,1% (TBS-T 0,1%) for 45 min, rinsed in distilled water and probed with the following primary antibodies diluted in TBS-T 0,1%, 0,5% BSA for 1 h at RT: rabbit anti-TUBGCP2 (1/2000, Homemade), rabbit anti-TPX2 (1 ug/ml, Homemade), mouse anti-acetylated tubulin (1:1000, Sigma, T7451), rabbit anti-β-tubulin (1:1000, Abcam, Ab6046). After primary antibody incubation and 3 washes of 5 min in TBS-T 0,1%, membranes were incubated for 45 min at RT with the following secondary

antibodies diluted in TBS-T0,1%, 0,5% BSA: goat anti-rabbit irDye 800CW (1:20000, Fisher Scientific, 10733944) and goat anti-mouse Alexa Fluor™ 680 (1:20000, Invitrogen, A21058) . After secondary antibody incubation, membranes were washed three times with TBS-T 0,1% and subjected to developing using the Oddysey infrared imaging system. The signal intensity of the γ-TuRC components in mitosis was measured using Fiji software, normalized to the tubulin signal intensity and represented in a scatter plot.

Structural Modelling of the *TUPBGCP2* missense mutation

The structural modeling of GCP2_E311K:GCP3 complex was carried out with the HADDOCK web server (https://haddock.science.uu.nl/services/HADDOCK2.2/ (Vangone et al., 2017) by using chains C and D of the γ-TuRC ring complex as a template (pdb id: 6v6s). A similar mutation modeling procedure was described in Dafsari *et al.* (Dafsari et al., 2019). To generate the electrostatic surfaces: (i) the relevant .pqr files were calculated with the http://server.poissonboltzmann.org/ server (Dolinsky et al., 2004), (ii) the acquired .pqr distributions were visualized with the APBS plugin of PyMOL.

Proteomic Profiling

Sample preparation and trypsin digestion

In total seven samples (fibroblasts) derived from 4 healthy controls (gender and age-matched) and from one TUBGCP patients (processed and measured in triplicates) were processed independently. After harvesting cells were lysed in 500 µL of lysis buffer (50 mM Tris-HCl (Applichem Biochemica A3452) (pH 7.8) 150 mM NaCl, 1 % SDS (Carl Roth CN30.1), and Complete Mini Roche 11873580001) and treated with Benzonaze (Sigma-Aldrich) for 30

minutes at 37°C in order to degrade the DNA. Then samples were centrifuged for 5 min at 4°C and 5000 *g*. Protein concentration of the supernatant was determined by BCA assay (ThermoFisher 23225) (according to the manufacturer's protocol) and cysteines were reduced with 10 mM of DTT (Roche 10708984001) by incubation at 56°C for 30 min. Next, the free thiol groups were alkylated with 30 mM IAA (Sigma-Aldrich I1149-25G) at room temperature (RT) in the dark for 30 minutes. Sample cleanup and proteolysis were performed using filter-aided sample preparation (FASP) as described previosuly (Mingirulli et al., 2020) . Briefly, 100 µg of protein was diluted 10-fold with freshly prepared 8M urea (Sigma-Aldrich)/100mM Tris-HCl (Applichem Biochemica) (pH 8.5) buffer and placed on a centrifugal device Nanosep 30 kDa Omega (Merck). The device was centrifuged at 13,800 g at RT for 20 min for all centrifugation steps. First, to eliminate residual SDS, three washing steps were carried out with 100 µL of 8M urea (Sigma-Aldrich) /100mM Tris-HCl (Applichem Biochemica) (pH 8.5). Then, for buffer exchange, the device was washed thrice with 100 µL of 50mM NH4HCO3 (Sigma-Aldrich S2889-250G) (pH 7.8). Next, 100 µL of proteolysis buffer comprising of trypsin (Promega) (1:25 w/w, protease to substrate), 0.2M GuHCl (Sigma-Aldrich) and 2mM CaCl2 ((Sigma-Aldrich)) in 50mM NH4HCO3 (Sigma-Aldrich) (pH 7.8), was added to the device and incubated at 37 °C for 14 h. Afterwards, the generated tryptic peptides were recovered by centrifugation with 50 µL of 50mM NH4HCO3 (Sigma-Aldrich) followed by 50 µL of ultra-pure water. Finally, peptides were acidified ((pH<3) by addition of 10% TFA (Biosolve) (v/v) and digests were quality-controlled in a reversed-phase HPLC as described previously (Mingirulli et al., 2020).

LC-MS/MS analysis

Samples (1 µg) were analyzed using an Ultimate 3000 nano RSLC system coupled to an LTQ Orbitrap Velos mass spectrometer (both Thermo Scientific). Peptides were preconcentrated

on a 75 µm x 2 cm C18 trapping column for 16 min using 0.1 % TFA (v/v) with a flow rate of 20 µl/min followed by separation on a 75 µm x 50 cm C18 main column (both Pepmap, Thermo Scientific) with a 130 min LC gradient ranging from 3-38 % ACN in 0.1 % FA (v/v) at a flow rate of 250 nl/min. MS survey scans were acquired in the Orbitrap from m/z 300 to 1500 at a resolution of 60,000 using the polysiloxane ion at *m/z* 371.101236 as lock mass. Theten most intense signals were subjected to collision induced dissociation (CID) in the ion trap taking into account a dynamic exclusion of 25 s. CID spectra were acquired with a normalized collision energy of 35 %. AGC target values were set to 10^6 for Orbitrap MS and 10^4 for ion trap MS^n scans.

Label free data analysis

Label free quantification of the acquired MS data was performed using the Progenesis LC-MS software from Nonlinear Dynamics (Newcastle upon Tyne, U.K.). Raw files were imported and the alignment of the MS runs was done automatically by the software by choosing one of the runs as reference. After peak picking, only features within retention time and m/z windows 0-130 min and 300-1500, respectively, and with charge states +2, +3 and +4 were considered for peptide statistics, analysis of variance (ANOVA) and principal component analysis (PCA). Spectra were exported as peak lists and searched against a concatenated target/decoy version of the human Uniprot database, (downloaded on 22.07.2015 containing 20273 target sequences) using Mascot 2.4 (Matrix Science), MS-GF+ and X!Tandem (version 2013.02.01.1) with the help of searchGUI 3.2.5 (Vaudel et al., 2011). Trypsin with a maximum of two missed cleavages was selected as enzyme. Carbamidomethylation of Cys was set as fixed, acetylation of protein N-terminus, oxidation of Met and phosphorylation of Ser/Thr/Tyr were selected as variable modifications. MS and MS/MS tolerances were set to 10 ppm and 0.5 Da, respectively. Search results were combined at a false discovery rate (FDR) of 1 % on the protein level using

the PeptideShaker software 1.4.0 (http://code.google.com/p/peptide-shaker/) and processed for re-import as peptide spectrum matches into Progenesis. Finally, 1681 proteins which were quantified from the non-conflicting features (i.e. only unique peptides) were exported. For all proteins, the normalized abundances from Progenesis were used to calculate standard deviations (SD) and coefficients of variation (CV) using all three replicates per condition. Only proteins with a p-value <0.05 and showing an average ratio <0.45 or >2.28 were considered as regulated.

TUBGCP2 expression in human skin fibroblasts was investigated by screening a protein/ spectral library covering 96,512 peptides referring to 8234 proteins expressed in these cells (Hentschel et al., preprint available DOI: 10.21203/rs.3.rs-48014/v1) for tryptic peptides unique for TUBGCP2.

Immunofluorescence studies on human skin fibroblasts

Immunofluorescence studies on human skin fibroblasts were carried out as following: cells were grown to a confluency of 60% on cover slips in a 24-well plate, washed twice with PBS, fixed with 4% formalin in PBS for 15 min and after the fixative was aspirated, cells were washed again twice with PBS. To quench the free aldehyde groups of the fixative, 10 mM NH_4Cl-PBS was added to the cells and incubated for 15 min. After fibroblasts were washed two times with PBS, they were permeabilized by adding 0.5 ml 0.1% Triton X-100 (in PBS) to each well (incubation for 10 min). Next, blocking was carried out by adding 100 µl 1% BSA made in PBS containing 0.1% Triton X-100 (v/v) to each well. Primary antibodies (α-CD63: ab8219, α-aB-Crystallin: sc-137129, α-Desmin: NCL-L-DES-DERII, α-PHGDH: GTX101948, Phalloidin: ab176753, α-Tenascin: AB19011) were diluted in 1% BSA blocking solution made in PBS containing 0.1% Triton X-100 (v/v) and incubated for one hour at room temperature. After

antibody solutions were aspirated and fibroblasts were washed with PBS containing 0.1% Triton X-100 secondary antibodies (Invitrogen Alexa488 & Alexa594) diluted 1:500 in 1% BSA blocking solution made in PBS containing 0.1% Triton X-100 (v/v) were added and incubated for one hour at room temperature. In the following step, antibody solutions were aspirated, and fibroblasts washed twice with PBS containing 0.1% Triton X-100. For mounting, 10 µl mounting medium (containing anti-fade reagent) was placed on the slide for each coverslip and coverslips were positioned at the centre of the slide. Excess of mountant was drawn with filter paper and mounting medium was solidified for two hours before samples were examined on microscope (Zeiss Axioplan).

Investigation of cellular fitness

To measure cellular metabolic activity as an indicator of cell viability, proliferation and cytotoxicity, the MTT-assay (Sigma, M5655-100MG) was applied to TUBGCP2-patient derived and control fibroblasts treated and non-treated with 80 µm L-serine (Sigma, S4311-25G), respectively. Viable cells contain NAD(P)H-dependent oxidoreductase enzymes which reduce the MTT to formazan; the insoluble formazan crystals are dissolved using a solubilization solution and the resulting coloured solution is quantified by measuring absorbance at 500-600 nanometers using a multi-well spectrophotometer (Tecan Infinity 200). Here, the darker the solution, the greater the number of viable, metabolically active cells. The assay was carried out according to manufacturer's specifications.

Supplementary Figures and Table

Supplementary Figure 1 (related to Figure 5) shows Violin plots depicting tissue/cell expression of TUBGCP2 from GTEx Portal (https://gtexportal.org/home/gene/TUBGCP2) **illustrate high expression in neuronal cell types, but also in cultured skin fibroblasts.**

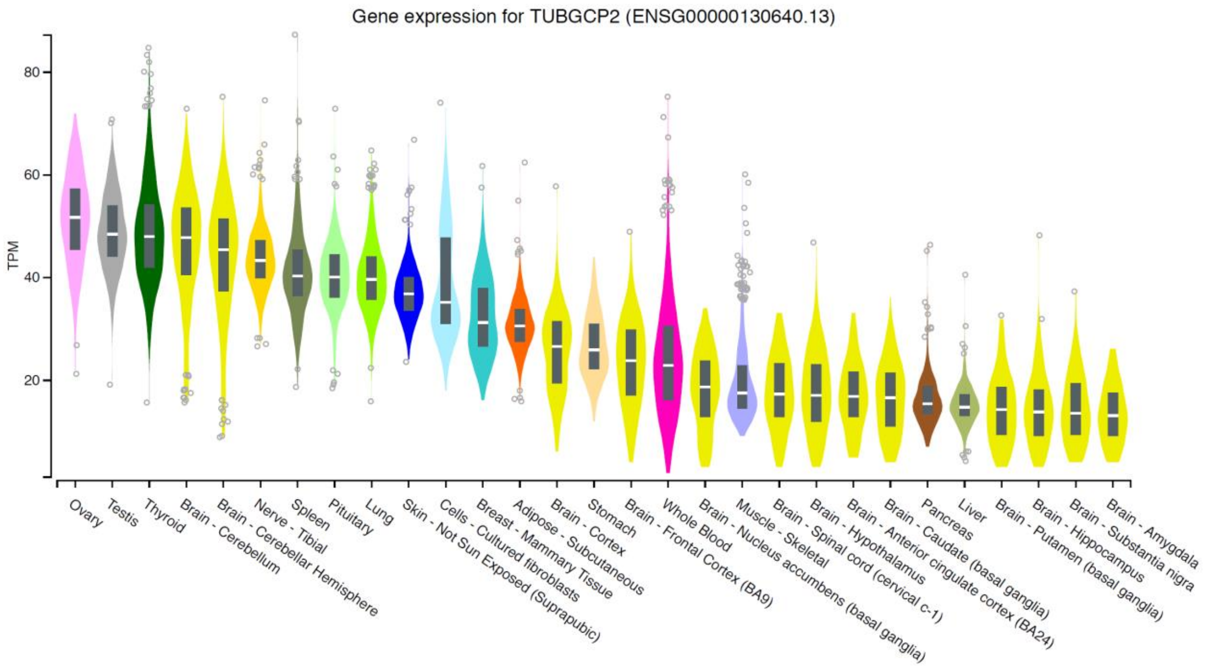

Supplementary Figure 2 (related to Figure 5). Protein targets of TUBGCP2 used in our proteomics studies (bold highlighted, p.E311K red).

>sp|Q9BSJ2|GCP2_HUMAN Gamma-tubulin complex component 2 OS=Homo sapiens OX=9606 GN=TUBGCP2 PE=1 SV=2

MSEFRIHHDVNELLSLLRVHGGDGAEVYIDLLQKNRTPYVTTTVSAHSAKVKIAEFSRTPEDFLKKYDELKSKNTR**NL**
DPLVYLLSKLTEDKETLQYLQQNAKERAELAAAAVGSSTTSINVPAAASKISMQELEELRK**QLGSVATGSTLQQSLEL**
KRKMLRDKQNKKNSGQHLPIFPAWVYERPALIGDFLIGAGISTDTALPIGTLPLASQESAVVEDLLYVLVGVDGRYVS
AQPLAGRQSR**TFLVDPNLDLSIR**ELVHR**ILPVAASYSAVTR**FIEEK**SSFEYGQVNHALAAAMR**TLVKEHLILVSQLEQ
LHRQGLLSLQKLWFYIQPAMRTMDILASLATSVDKGECLGGSTLSLLHDRSFSYTGDSQAQELCLYLTK**AASAPYFEV**
LEKWIYRGIIHDPYSEFMVEEHELRKER**IQEDYNDKYWDQRYTIVQQQIPSFLQK**MADKILSTGKYLNVVR**ECGHD**
VTCPVAKEIIYTLKERAYVEQIEKAFNYASKVLLDFLMEEKELVAHLRSIKRYFLMDQGDFFVHFMDLAEEELRKPVED
ITPPRLEALLELALR**MSTANTDPFKDDLK**IDLMPHDLITQLLRVLAIETKQEKAMAHADPTELALSGLEAFSFDYIVKW
PLSLIINRKALTRYQMLFRHMFYCKHVERQLCSVWISNKTAKQHSLHSAQWFAGAFTLRQRMLNFVQNIQYYMM
FEVMEPTWHILEKNLKSASNIDDVLGHHTGFLDTCLKDCMLTNPELLKVFSKLMSVCVMFTNCMQKFTQSMK**LDG**
ELGGQTLEHSTVLGLPAGAEERARKELARKHLAEHADTVQLVSGFEATINKFDKNFSAHLLDLLAR**LSIYSTSDCEHG**
MASVISRLDFNGFYTERLERLSAERSQKATPQVPVLRGPPAPAPRVAVTAQ

Supplementary Table 1 (related to Figure 5) shows the list of differentially regulated proteins in *TUBGCP2* mutant fibroblasts.

Uniprot Accession #	Protein name	Gene name	#Peptides	Fold change	Anova (p)	Protein function
Q9UKX5	Integrin alpha-11	*ITA11*	4	4.21	<0.0005	Receptor for collagen
Q08431	Lactadherin	*MFGM*	2	3.58	<0.0005	Essential role in neurons in Aβ-induced phagoptosis
P52943	Cysteine-rich protein 2	*CRIP2*	2	3.46	<0.0005	Invadopodia actin bundling factor
P02511	Alpha-crystallin B chain	*CRYAB*	8	3.27	<0.0005	Chaperone-like activity, preventing aggregation of various proteins
P17661	Desmin	*DESM*	4	3.24	<0.0005	Act as a (sarcomeric) microtubule-anchoring protein: specifically associates with detyrosinated tubulin-alpha chains, leading to buckled microtubules
Q6UVK1	Chondroitin sulfate proteoglycan 4	*CSPG4*	15	2.79	<0.0005	Inhibits neurite outgrowth and growth cone collapse during axon regeneration; cell surface receptor for collagen alpha 2(VI)
Q8TAD7	Overexpressed in colon carcinoma 1 protein	*OCC1*	3	2.74	<0.0005	
Q14699	Raftlin	*RFTN1*	2	2.64	<0.0005	Involved in protein trafficking; mediates internalization of TLR4 to endosomes in dendritic cells
P55290	Cadherin-13	*CAD13*	6	1.40	<0.0005	Acts as a negative regulator of neural cell growth
O75326	Semaphorin-7A	*SEM7A*	4	1.35	<0.0005	Plays an important role in integrin-mediated signaling and functions; promotes axon growth in the embryonic olfactory bulb. Promotes attachment, spreading and dendrite outgrowth
Q14108	Lysosome membrane protein 2	*SCRB2*	3	2.38	<0.0005	Lysosomal receptor for glucosylceramidase (GBA) targeting
Q15149	Plectin	*PLEC*	219	2.34	<0.0005	Interlinks intermediate filaments with microtubules and microfilaments and anchors intermediate filaments to desmosomes or hemidesmosomes. Could also bind muscle proteins such as actin to membrane complexes in muscle
Q8TF66	Leucine-rich repeat-containing protein 15	*LRRC15*	1	10.66	<0.0005	Promotes osteogenic differentiation of mesenchymal stem cells
O94907	Dickkopf-related protein 1	*DKK1*	1	7.74	<0.0005	Inhibit Wnt regulated processes such as antero-posterior axial patterning, somitogenesis and eye formation; in adults implicated in bone formation cancer and Alzheimer disease
Q9Y6U3	Adseverin	*ADSV*	1	4.10	<0.0005	Ca^{2+}-dependent actin filament-severing protein that has a regulatory function in exocytosis by affecting the organization of the microfilament network underneath the plasma membrane
Q96LJ7	Dehydrogenase/reductase SDR family member 1	*DHRS1*	1	3.85	<0.0005	NADPH-dependent reductase that is able to catalyse the in vitro reductive conversion of some steroids
O94919	Endonuclease domain-containing 1 protein	*ENDOD1*	1	3.20	<0.0005	Act as a DNase and a RNase

Q9ULH0	Kinase D-interacting substrate of 220 kDa	*KDIS*	1	3.11	<0.0005	Plays a role in nerve growth factor (NGF)-induced recruitment of RAPGEF2 to late endosomes and neurite outgrowth. May play a role in neurotrophin- and ephrin-mediated neuronal outgrowth and in axon guidance during neural development and in neuronal regeneration
Q5JRX3	Presequence protease (mitochondrial)	*PREP*	1	2.78	<0.0005	Metalloendopeptidase of the mitochondrial matrix that functions in peptide cleavage and degradation
Q96HC4	PDZ and LIM domain protein 5	*PDLI5*	1	2.76	0.01	Actin binding protein which plays an important role in the heart development by scaffolding PKC to the Z-disk region; overexpression promotes the development of heart hypertrophy
Q9BRK3	Matrix-remodeling-associated protein 8	*MXRA8*	1	2.74	<0.0005	Modulates activity of various signaling pathways, probably via binding to integrin
P16402	Histone H1.3	*H1-3*	1	2.67	<0.0005	Binds to linker DNA between nucleosomes and acts as a regulator of individual gene transcription through chromatin remodeling
Q14956	Transmembrane glycoprotein NMB	*GPNMB*	1	2.65	<0.0005	Activator of the ERK1/2 and Akt pathways toward the prevention of build-up of TDP-43 aggregates
P18827	Syndecan-1	*SDC1*	1	2.50	0.01	Cell surface proteoglycan that links the cytoskeleton to the interstitial matrix and regulates exosome biogenesis
O75521	Enoyl-CoA delta isomerase 2 (mitochondrial)	*ECI2*	1	2.30	<0.0005	Isomerizes both, 3-cis and 3-trans double bonds into the 2-trans form in a range of enoyl-CoA species
P48061	Stromal cell-derived factor 1	*CXCL12*	1	2.29	0.02	Induces migration of oligodendrocyte precursor cells through activated ERK and AKT pathways
P02461	Collagen alpha-1(III) chain	*CO3A1*	19	0.45	<0.0005	Involved in regulation of cortical development; major ligand of ADGRG1 in the developing brain and binding to ADGRG1 inhibits neuronal migration and activates the RhoA pathway
Q15063	Periostin	*POSTN*	3	0.45	<0.0005	Induces cell attachment and spreading and plays a role in cell adhesion
P55957	BH3-interacting domain death agonist	*BID*	1	0.45	0.01	Initiates apoptosis
Q7Z434	Mitochondrial antiviral-signaling protein	*MAVS*	1	0.45	<0.0005	MAVS signaling activation causes induction of autophagic activation in brain
P40261	Nicotinamide N-methyltransferase	*NNMT*	6	0.45	<0.0005	Protectant against neurotoxin-mediated cell death; increased expression promotes neurite branching, synaptophysin expression and dopamine accumulation and release
O43175	D-3-phosphoglycerate dehydrogenase	*PHGDH*	16	0.42	<0.0005	Modulates first step of the phosphorylated L-serine biosynthesis pathway
Q9Y613	FH1/FH2 domain-containing protein 1	*FHOD1*	1	0.42	<0.0005	Contributes to the coordination of microtubules with actin fibers and plays a role in cell elongation
O14495	Lipid phosphate phosphohydrolase 3	*LPP3*	3	-1.28	<0.0005	Involved in cell adhesion and in cell-cell interactions; lack in embryonic stem cells compromises neuronal differentiation and neurite outgrowth

Q02952	A-kinase anchor protein 12	*AKA12*	17	-1.33	<0.0005	Anchoring protein that mediates the subcellular compartmentation of protein kinase A (PKA) and protein kinase C (PKC)
P22692	Insulin-like growth factor-binding protein 4	*IGFBP4*	2	-1.37	<0.0005	Inhibits proliferation and promotes differentiation of neural progenitor cells
P60174	Triosephosphate isomerase	*TPIS*	20	-1.37	<0.0005	Reduced function of this protein triggers neuronal death
Q9BRA2	Thioredoxin domain-containing protein 17	*TXNDC17*	3	-1.38	<0.0005	Disulfide reductase; modulates TNF-alpha signaling and NF-kappa-B activation
P10620	Microsomal glutathione S-transferase 1	*MGST1*	3	-1.39	<0.0005	Conjugation of reduced glutathione to a wide number of exogenous and endogenous hydrophobic electrophiles
Q16647	Prostacyclin synthase	*PTGIS*	6	-1.41	<0.0005	Catalyzes the isomerization of prostaglandin H2 to prostacyclin
P24821	Tenascin	*TENA*	24	-1.52	0,01	Extracellular matrix protein implicated in guidance of migrating neurons as well as axons during development, synaptic plasticity as well as neuronal regeneration. Promotes neurite outgrowth from cortical neurons
P52209	6-phosphogluconate dehydrogenase, decarboxylating	*PGD*	8	-1.70	<0.0005	Catalyzes the oxidative decarboxylation of 6-phosphogluconate to ribulose 5-phosphate and CO_2
P48681	Nestin	*NEST*	8	-1.70	<0.0005	Required for brain and eye development. Promotes the disassembly of phosphorylated vimentin intermediate filaments (IF) during mitosis and may play a role in the trafficking and distribution of IF proteins and other cellular factors to daughter cells during progenitor cell division. Required for survival, renewal and mitogen-stimulated proliferation of neural progenitor cells
Q15392	Delta(24)-sterol reductase	*DHC24*	2	0.29	<0.0005	Protects cells from oxidative stress; protects against amyloid-beta peptide-induced apoptosis
Q93062	RNA-binding protein with multiple splicing	*RBPMS*	2	-2.29	<0.0005	Acts as a coactivator of transcriptional activity
P53634	Dipeptidyl peptidase 1	*CATC*	3	-2.43	<0.0005	Activates serine proteases such as elastase, cathepsin G and granzymes A and B; can also activate neuraminidase
P24593	Insulin-like growth factor-binding protein 5	*IGFBP5*	2	-4.69	<0.0005	Inhibitory binding protein for insulin-like growth factor 1; overexpression leads to motor axonopathy and sensory deficits in mice
P54826	Growth arrest-specific protein 1	*GAS1*	1	0.03	0.04	Specific growth arrest protein involved in growth suppression; blocks entry to S phase; promotes neurite outgrowth
Q8NDI1	EH domain-binding protein 1	*EHBP1*	1	-2.00	0.01	Plays a role in actin reorganization; links clathrin-mediated endocytosis to the actin cytoskeleton; May act as Rab effector protein and play a role in vesicle trafficking
P26022	Pentraxin-related protein PTX3	*PTX3*	1	-6.20	<0.0005	Plays a protective role in seizure-induced neurodegeneration

References

Dafsari, H.S., Sprute, R., Wunderlich, G., Daimaguler, H.S., Karaca, E., Contreras, A., Becker, K., Schulze-Rhonhof, M., Kiening, K., Karakulak, T., *et al.* (2019). Novel mutations in KMT2B offer pathophysiological insights into childhood-onset progressive dystonia. J Hum Genet *64*, 803-813.

Dolinsky, T.J., Nielsen, J.E., McCammon, J.A., and Baker, N.A. (2004). PDB2PQR: an automated pipeline for the setup of Poisson-Boltzmann electrostatics calculations. Nucleic Acids Res *32*, W665-667.

Mingirulli, N., Pyle, A., Hathazi, D., Alston, C.L., Kohlschmidt, N., O'Grady, G., Waddell, L., Evesson, F., Cooper, S.B.T., Turner, C., *et al.* (2020). Clinical presentation and proteomic signature of patients with TANGO2 mutations. J Inherit Metab Dis *43*, 297-308.

Vangone, A., Rodrigues, J.P., Xue, L.C., van Zundert, G.C., Geng, C., Kurkcuoglu, Z., Nellen, M., Narasimhan, S., Karaca, E., van Dijk, M., *et al.* (2017). Sense and simplicity in HADDOCK scoring: Lessons from CASP-CAPRI round 1. Proteins *85*, 417-423

Vaudel, M., Barsnes, H., Berven, F.S., Sickmann, A., and Martens, L. (2011). SearchGUI: An open-source graphical user interface for simultaneous OMSSA and X!Tandem searches. Proteomics *11*, 996-999.
